# Influence of Fluorine-Containing Monomer Content on the Hydrophobic and Transparent Properties of Nanohybrid Silica Polyacrylate Coating Materials

**DOI:** 10.3390/ma14154261

**Published:** 2021-07-30

**Authors:** Chih-Ming Huang, Her-Yung Wang, Sing-Yuan Fang, Wein-Duo Yang

**Affiliations:** 1 Department of Chemical and Materials Engineering, National Kaohsiung University of Science and Technology, Kaohsiung 807, Taiwan; color.jackhuang@gmail.com (C.-M.H.); v27946@gmail.com (S.-Y.F.); 2 Department of Civil Engineering, National Kaohsiung University of Science and Technology, Kaohsiung 807, Taiwan; wangho@nkust.edu.tw

**Keywords:** fluorine-containing, polyacrylate, nanohybrid, hydrophobic properties, visible light

## Abstract

Nanosilica-modified, fluorine-containing polyacrylate hybrid coating materials, consisting of dodecafluoroheptyl methacrylate (DFMA), methyl methacrylate (MMA), 2-ethyl hexyl acrylate (2-EHA), 3-(trimethoxysilyl) propyl methacrylate (KH-570), and tetraethylorthosilicate (TEOS), are synthesized successfully by free radical polymerization and the sol–gel process. It is revealed that the content of the fluorine-containing polyacrylate hybrid coating materials from DFMA monomers significantly improves the properties of the films. The polyacrylate coating film prepared with a weight ratio of DFMA/MMA at 1:5 exhibits the largest water contact angle of 105.4°, which demonstrates that DFMA can effectively improve the hydrophobicity of the coating film. Moreover, the silicon coupling agent (KH-570) is used to graft silica with acrylate. Spherical in shape, the surface morphology of the nanohybrid film exhibits a core–shell structure, which increases the surface roughness and enhances the hydrophobic properties. The as-prepared fluorine-containing nanohybrid silica polyacrylate film possesses a high transmittance of 89–97% in the visible light region, indicating its potential as a very attractive solution in many practical areas.

## 1. Introduction

When observing the self-cleaning lotus in nature, the phenomenon of hydrophobicity/super-hydrophobicity is revealed [1]. The definition of hydrophobicity is that the water contact angle (WCA, θ) θ > 90° of the surface is hydrophobic, and the WCA of the surface θ < 90° is hydrophilic [2]. When the surface possesses a WCA θ > 150° and a sliding angle (SA) less than 10°, it is called a superhydrophobic surface [3]. The most important factors that affect the hydrophobic properties of the surface are increases in the surface roughness and decreases in the surface energy [4,5,6]. Thus, the methods applied for manufacturing a hydrophobic surface are etching [7], lithography [8,9], chemical vapor deposition [10], chemical and electrochemical deposition [11,12], anodizing [13,14], plasma treatment [15,16,17], sandblasting [18], phase separation [19], the sol–gel process [20,21], and layer-by-layer assembly [22,23], among others. However, most of the above methods require multiple steps to complete, or require expensive equipment; they are not economical in practice for industrial applications.

Polyacrylate resin has been characterized by high transparency [24], excellent adhesion [25], diverse monomer sources [26], and high mechanical strength [27], and is the most attractive polymer. However, owing to its insufficient thermal stability, weather resistance, and anti-corrosion properties, it is limited in its practical applications [28].

SiO_2_ possesses a unique structure and excellent physical–chemical properties, and is widely used to enhance the physical and chemical properties of polymer materials [29]. Many silanol groups on the surface of SiO_2_ not only exhibit good wettability, but also interact with the interface of the mixed resin.

In order to improve the above-mentioned shortcomings of polyacrylate resins, SiO_2_ nanoparticles with “high surface energy and high specific surface area” are often mixed with the resin, but SiO_2_ is prone to agglomeration, which leads to phase separation in the organic–inorganic composite. Therefore, the combination of functionalized SiO_2_ nanoparticles and polyacrylate is an important route to enhance the dispersion and compatibility of the material [30,31,32].

Recently, a few articles have reported the use of monomer-containing fluorine for applications in modifying the structure of acrylate [33,34]. The modified fluorine-based acrylate resin has a low surface energy on the surface of the coating film, as a result of improvements to the hydrophobicity and thermal stability [35]. In addition, in the synthesis of organic–inorganic composite materials, the surface roughness, water contact angle (WCA), and weather resistance of the composite coating film can be also enhanced [36]. Generally, 3-(trimethoxysilyl) propyl methacrylate (KH-570) or 3-(triethoxysilyl) propyl methacrylate (MPS) is commonly used as a silicon coupling agent for the grafting bridge of organic–inorganic composite materials [37,38]. Tetraethylorthosilicate (TEOS) is utilized as the precursor of the inorganic structure (SiO_2_) through the sol–gel route to graft with the silicon coupling agent (KH-570). After grafting, organic–inorganic cross-linking is achieved, so as to increase the mechanical properties and adhesion of the coating film [39,40]. Increasing the surface roughness can improve the hydrophobic properties of the coating film, but it can also cause light scattering and decrease the transparency property in the visible light [41].

In 2019, a fluorinated/silanized polyacrylate amphiphilic polymer was reported by Zhu et al. [42]. It has been revealed that, by changing the content of 2-perfluorooctylethyl methacrylate (FMA), the surface wettability and protein adsorption performance of the as-prepared polyacrylate film can be significantly regulated. Yu et al. [43] reported a nano-SiO_2_/fluorinated polyacrylate polyurethane coating film showing both good hydrophobicity and good anti-aging properties. Both the chemical resistance and the mechanical properties of the composite coating were improved by the synergistic effect of the fluorine segments cross-linked in the polymer and nano-SiO_2_ particles. Moreover, the synthesis of cellulose nanocrystal-armored fluorinated polyacrylate latexes via Pickering emulsion polymerization was reported by Li et al. [44]. It was shown that the water–oil repellent and mechanical properties of the film could be improved as the content of the used Pickering stabilizer increased. The thermal analysis also exhibited an enhancement of the thermal properties of latex film upon increasing the stabilized content.

Wang et al. [45] published the study of a waterborne polyurethane prepolymer modified with styrene, butyl acrylate, glycidyl methacrylate, and dodecafluoroheptyl methacrylate through a soap-free emulsion polymerization method to obtain the cross-linked, fluorinated acrylate-modified waterborne polyurethane coating, which possessed the characteristics of an external hydrophobic surface constructed by long fluorinated side-chains and an internal double cross-linking system. The preparation of an amphiphilic Janus SiO_2_/fluorinated polyacrylate latex film and its application as a hydrophobic fabric agent was reported by Lyu et al. [46]. The water contact angle of the as-prepared fabric increased from 21.4° to 140.2°, demonstrating a great improvement in hydrophobicity and enhancing the water resistance of the latex film.

Additionally, Shao et al. improved the mechanical properties of fluorine-containing acrylic copolymers obtained by mixing emulsifiers by a semi-continuous seeded emulsion polymerization method [47]. Although the eco-friendly and biodegradable emulsifiers were used to reduce environmental pollution, the hydrophobic effect and conversion rate of the copolymer decreased. To the best of our knowledge, there are a few reports that have studied polyacrylate resin as prepared by fluorine-containing acrylate monomer in order to improve its hydrophobic and anti-corrosion properties. However, there are few studies that focus on the influence of the fluorine content on the hydrophobicity and transparent properties in the visible region of the polyacrylate-SiO_2_ material. In order to adapt to the application of polyacrylate with a high light transmittance in an outdoor environment, this issue is important.

In this study, dodecafluoroheptyl methacrylate (DFMA), methyl methacrylate (MMA), and 2-ethyl hexyl acrylate (2-EHA) were utilized as the monomers, and (3-(trimethoxysilyl) propyl methacrylate, KH-570) was used as the silicon coupling agent to synthesize acrylate resin. Then, tetraethylorthosilicate (TEOS) was added by the sol–gel route in order to prepare nanohybrid silica polyacrylate coating materials. By changing the weight ratios of MMA/DFMA, high hydrophobicity and a high light transparency in the polyacrylate films were obtained. The influence of the hydrophobicity, surface roughness, thermal properties, and light transmittance of the coating film surface by the various weight ratios of MMA/DFMA was investigated. The as-prepared polyacrylate-SiO_2_ nanohybrid material can be applied to the coating of 3C electronic products, vehicles, and construction industries.

## 2. Experimental

### 2.1. Materials

Analytical-grade chemicals were used as received, without any further purification. Dodecafluoroheptyl methacrylate (97.0% purity, Tokyo Chemical, Tokyo, Japan), methyl methacrylate (MMA, 99.0% purity, Alfa Aesar, Haverhill, MA, USA), 2-ethylhexyl acrylate (2-EHA, 98.0% purity, Sigma-Aldrich, Saint louis, MO, USA), tetrahydrofuran (THF, 99.8% purity, Mallinckrodt Chemical, Staines-upon-Thames, England), azobisisbutyronitrile (AIBN, 99.0%, Aencore, Whitehorse, Australia), tetraethylorthosilicate (TEOS, 98.0% purity, Sigma-Aldrich,), 3-(trimethoxysilyl) propyl methacrylate (KH-570, 98.0% purity, Sigma-Aldrich,), and hydrochloric acid (HCl, 37.0% purity, Aencore) were used for the preparation of the nanohybrid silica polyacrylate coating material.

### 2.2. Preparation of Organic–Inorganic Grafting Silica Polyacrylate Material

First, MMA, DFMA, and 2-EHA were used as the monomers and KH-570 was used as the silicon coupling agent; the starting materials were weighed by the designed amounts and mixed well with 40 g THF solvent, which was placed in a 250 mL round bottom flask equipped with a reflux condenser. Then, the flask placed in an oil bath was heated up to 70 °C and kept at this temperature under a nitrogen atmosphere at a stirring speed of 200 rpm. The initiator, 0.02 g of AIBN, was added to the previously prepared mixed solution, and polymerization was carried out for 5 h at a constant temperature of 70 °C. After the polymerization reaction was completed, the product was cooled to room temperature.

An amount of 1.0 g of TEOS was taken and placed in a flask, and then mixed with HCl aqueous solution (pH approximate at 1) used as a catalyst to progress the hydrolysis and polycondensation for obtaining a Si-sol by the sol–gel route, in advance.

Finally, the as-obtained Si-sol was dissolved in 5 g of THF and dropped into the acrylic-based copolymer by peristaltic pump at a rate of 2 mL/min, and stirred continuously for 16 h for the grafting for the reaction of KH-570 contained in reaction of polyacrylate and SiO_2_ to obtain an organic–inorganic silica nanohybrid polyacrylate material. The recipes of the starting materials for the preparation of the nanohybrid silica polyacrylate coating material are shown in Table 1. In this preparation, the weight ratio of DFMA/MMA is taken as the primary variable. In order to keep the weight of the as-prepared polyacrylate fixed, the ratio of each component monomer was designed to change the weight of DFMA and MMA monomers at the same time, but the total weight of DFMA and MMA remained unchanged. In addition, changing both DFMA and MMA at the same time, it is easier to maintain the original stoichiometry of the composition of each monomer in the prepared polymer, avoiding excessive composition deviation of the as-prepared polyacrylate.

### 2.3. Preparation of the Silica-Polyacrylate Coating Film

A doctor blade was used to prepare a coating film for the as-obtained silica-polyacrylate resin. The resin was coated and heat-cured at 75 °C for 2 h on a 10 cm × 10 cm copper substrate to remove water and residual solvents. Then, a dried silica nanohybrid polyacrylate coating film with a thickness of approximately 0.5 mm was obtained.

### 2.4. Characterization

The organic functional groups of the as-prepared nanohybrid silica polyacrylate materials were characterized by Fourier transform infrared spectroscopy (FTIR; BIO-RAD, FIS-165, Hercules, America). Differential scanning calorimetry (DSC, TA Instruments MDS2920, Milford, CT, USA) and a thermogravimetric analyzer/differential thermal analyzer (TGA/DTA, TA Instruments Q600, Milford, CT, USA) were utilized to reveal the thermal stability of the obtained materials.

The surface structure and morphology of the as-prepared polyacrylate-SiO_2_ films were analyzed by field emission scanning electron microscopy (FESEM, JEOL JSM-7401F, Tokyo, Japan) and transmission electronic microscopy (TEM, JEOL, JEM-3010). For TEM characterization, the as-prepared organic‒inorganic grafting acrylic-based resin was diluted by THF solvent to obtain a solid content approximate 1 wt.% of diluted acrylic-based resin. Then, the diluted resin was dropped on a 400 mesh carbon-plated copper grid and allowed to dry, and a film coated on the copper grid was obtained for TEM analysis. Moreover, the surface roughness of the material was analyzed by atomic force microscopy (BASO-AFM, Guangzhou, China).

The contact angle is defined as the angle between the solid surface and a tangent to the drop-surface. The water contact angles of the as-prepared nanohybrid Si-polyacrylate films were determined using a contact angle goniometer (NBSI, OSA60, Ningbo, China). Ultraviolet–visible–near-infrared DRS spectroscopy (UV–vis–NIR) (JASCO, V-670, Tokyo, Japan) was utilized to characterize the transparent property of the as prepared coating films in the UV–visible light region.

## 3. Results and Discussion

### 3.1. Preparation of Nanohybrid Silica Polyacrylate Coating Materials

**Figure 1:**
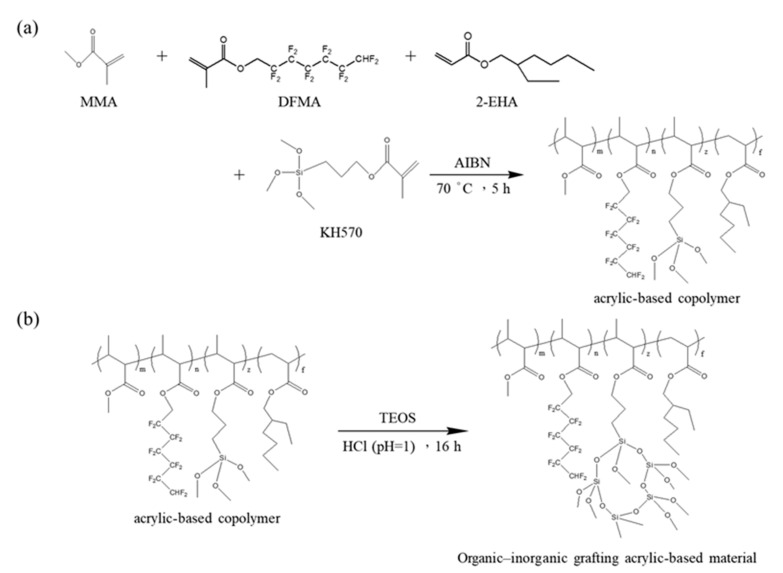
Copolymerization reactions of the organic‒inorganic nanohybrid silica polyacrylate materials: (**a**) the schematic of the preparation of acrylic-based copolymer, (**b**) the schematic of organic‒inorganic grafting acrylate-SiO_2_ resin.

MMA, DFMA, and 2-EHA were used as the monomers and KH-570 was used as the silicon coupling agent, and the acrylic-based copolymer was prepared in THF solvent by a free radical polymerization. Figure 1 indicates the copolymerization reactions of the organic–inorganic nanohybrid silica polyacrylate materials. The schematic of the preparation of acrylic-based copolymer is shown in Figure 1a.

During the preparation process, THF was used as the solvent. THF solvent can uniformly dissolve MMA, DFMA, 2-EHA, and KH570 monomers, allowing to obtain a uniform solution. Therefore, the acrylic-based copolymer (resin) obtained has excellent fluidity. Observing the prepared copolymer, it is transparent, colorless, and has good fluidity. The fluorinated polyacrylate copolymer obtained at this stage has disordered molecular chains. This diluted resin is very beneficial to the preparation of the next stage of organic‒inorganic grafting acrylic-based material.

TEOS undergoes hydrolysis and condensation reactions to obtain silica-sol from the sol‒gel route. The pH of the reaction is an important variable. In this study, under acidic condition (pH at approximately 1), the hydrolysis and condensation are slow, and the prepared silica-sol is transparent, which is favorable to the grafting reaction of organic‒inorganic hybrids. Nagarale et al. [48] pointed out that, in the sol‒gel reaction of TEOS, irregular linear transparent sols are easily formed under acid catalysts, and uniform white large granular silica precipitates are formed under alkaline catalysts. The schematic of the organic‒inorganic grafting acrylate-SiO_2_ resin is shown in Figure 1b. When the silica sol is grafted with the fluorinated acrylate copolymer, the Si-OH on the silica-sol will be covalently bonded with the silicon coupling agent (KH-570) contained in the as-prepared copolymer to obtain an organic‒inorganic grafting acrylic-based resin. The resin prepared is almost colorless and transparent.

After drying at 75 °C for 2 h, the THF solvent and residual water were removed, and the film thickness was about 0.5 mm. It is not necessary to add a curing agent for the preparation of an organic‒inorganic grafting acrylate-SiO_2_ coating film, which can be obtained by heat treatment only. During the drying process, there is not a violent exothermal reaction, and the adhesion between the coating film and the copper substrate is fine.

In the preparation of nanohybrid silica polyacrylate coating materials, it is revealed that, the higher the weight ratios of DFMA/MMA, the slower the evaporation of THF solvent and water, and the slower the film formation. This is because, in the process of heating and drying, the hydrophobic segments of the copolymer will move to the surface of the coating film, and the hydrophilic segments will be below the surface. THF and water are miscible and the Si-OH produced by the sol‒gel reaction is hydrophilic; therefore, the THF solvent and water under the coating film evaporate slowly, meaning that the film formation will be slower [49].

### 3.2. Physical Properties of the As-Prepared Nanohybrid Silica Polyacrylate Material

**Figure 2:**
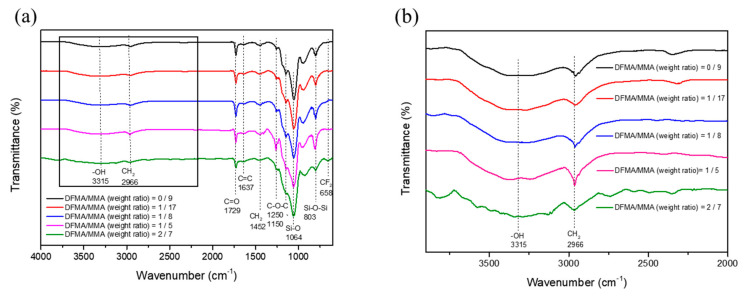
The Fourier transform infrared spectroscopy (FTIR) spectrum analysis of the prepared SiO_2_-polyacrylate material. (**a**) wavenumber range from 600 cm^−1^ to 4000 cm^−1^ and (**b**) a partial enlargement of (**a**).

Figure 2 shows the FTIR spectrum analysis of the prepared SiO_2_-polyacrylate material. The figure shows that the absorption peak at around 1729 cm^−1^ is attributed to the stretching vibration of the characteristic peak of C=O [50]. The characteristic bands of -CH_2_ are present at 1452 cm^−1^ and 2996 cm^−1^ [50]. The C-O-C asymmetric characteristic peak in MMA, DFMA, and 2-EHA is identified at the peak of 1250 cm^−1^. Moreover, the characteristic band at 658 cm^−1^ is assigned to be the characteristic of -CF_2_ [51]. When the DFMA/MMA weight ratio increases, the absorption peaks at 1150 cm^−1^ and 1236 cm^−1^ increase. The above two peaks are the overlap of C-O-C absorption and the absorption of the stretching vibration of C-F from DFMA [52,53].

The sol–gel method was used to graft SiO_2_ on the acrylate copolymer, and it was observed that the acrylic-based film possesses absorption bands at 803 cm^−1^ and 1064 cm^−1^, which are assigned to the Si-O-Si asymmetric characteristic peak and Si-O absorption characteristic peak in SiO_2_, respectively [54]. In addition, the small amount of the silicon coupling agent (KH-570) utilized in the sample preparation and the C=O group also existed in MMA, DFMA, and 2-EHA; therefore, it is impossible to clearly identify the characteristics of KH-570 from FTIR analysis [34]. Figure 2b is a partial enlargement of Figure 2a, and a broad characteristic band is observed at approximately 3315 cm^−1^. This may be attributed to the stretching vibration band of the -OH group, which did not undergo a condensation reaction with KH-570 after the hydrolysis of TEOS during the sol–gel process.

**Figure 3:**
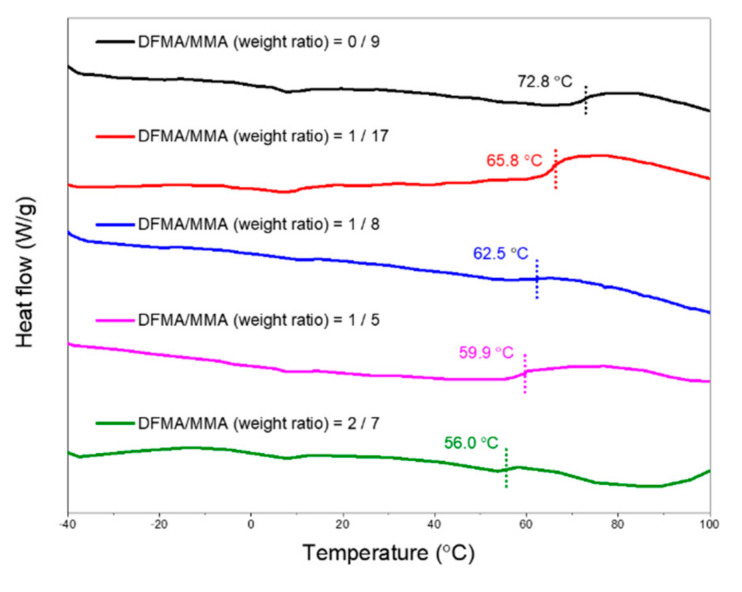
Differential scanning calorimetry (DSC) analysis of the as-prepared nanohybrid silica polyacrylate materials prepared by different dodecafluoroheptyl methacrylate (DFMA)/methyl methacrylate (MMA) weight ratios.

Figure 3 shows the DSC analysis of the as-prepared nanohybrid silica polyacrylate materials prepared by different DFMA/MMA weight ratios. The results show that the glass transition temperature (T_g_) of the acrylate copolymer, obtained without adding the fluorine-containing monomer (weight ratio of DFMA/MMA at 0:9), is 72.8 °C. As the weight ratio of DFMA/MMA increases, the T_g_ of the resulting copolymerized material shows a downward trend. When the weight ratio of DFMA/MMA is 1:5 (1.5:7.5), the T_g_ of the obtained acrylate-SiO_2_ material drops to 56.0 °C. The T_g_ of the homopolymer (PDFMA) polymerized from DFMA monomer is approximately 60 °C [54,55], which is lower than the T_g_ (105 °C) of PMMA obtained from the MMA monomer. Therefore, when the weight ratios of DFMA/MMA increase, the T_g_ of the as-prepared polyacrylate-SiO_2_ materials decreases.

**Figure 4:**
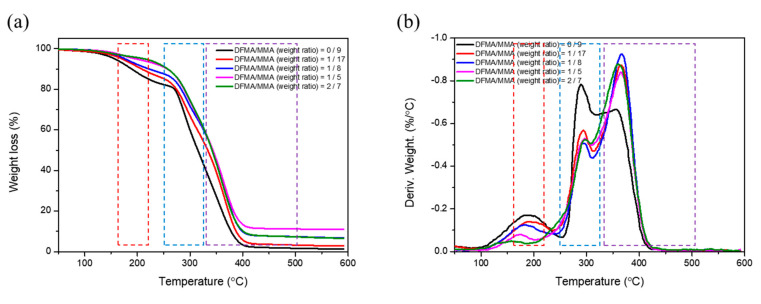
Thermogravimetric analyzer/differential thermal analyzer (TGA/DTA) analyses of the as-prepared nanohybrid silica polyacrylate. (**a**) TGA analysis and (**b**) DTA analysis.

Figure 4 shows the TGA/DTA analyses of the as-prepared nanohybrid silica polyacrylate. It is observed that the thermal decomposition curves of the material by TGA (Figure 4a) can be divided into three stages. The first stage is 170–210 °C and exhibits a weight loss of about 5–10%; this involves the unreacted monomers or impurities decomposed in the copolymer, resulting in thermogravimetric loss. The second stage is 230–310 °C and exhibits a weight loss of approximately 30–40%. The intermediate temperature peak is usually attributed to the cleavage of the double bond of the hydrocarbon group (-CH=CH_2_) in the acrylic copolymer. The third stage is 310–500 °C, exhibiting a weight loss of about 45–55%. This is the most important stage of the as-prepared SiO_2_-polyacrylate material during thermal pyrolysis. It is the random pyrolysis of the overall copolymer that causes the thermogravimetric loss. The TGA results are similar to the thermal decomposition of PMMA, as studied by Manring et al. [56].

However, it can be predicted from the TGA analysis from Figure 4 that the result of the decomposition starts at near the same temperature for all samples. Obviously, from the weight loss curve of TGA, the slopes of the curves are between approximately 200 °C and 400 °C for the five samples. The result reveals that the samples prepared by weight ratios of DFMA/MMA of 2:7 and 1:5 have slightly lower slopes (the two are almost the same) than the other samples, which means that the fluorine-containing compound (DFMA) slightly increased the stability of the as-prepared polyacrylate at the same temperature in the temperature range of 200 °C–400 °C. In particular, polyacrylate made from DFMA is more stable than that of material made without it, observed from the comparison of slope from weight loss curves.

Moreover, according to the DTA analysis (Figure 4b), the results show that, when the polyacrylate-SiO_2_ material is prepared without adding DFMA (weight ratio of DFMA/MMA = 0:9), the fraction of low-temperature pyrolysis (the second stage: 230–310 °C) is higher than those with added DFMA. On the contrary, when the weight ratios of DFMA/MMA increase, the fraction of low-temperature pyrolysis is much lower, while the fraction of high-temperature pyrolysis (third stage: 310–500 °C) increases.

### 3.3. Morphology Analyses of the As-Prepared Nanohybrid Silica Polyacrylate Film

**Figure 5:**
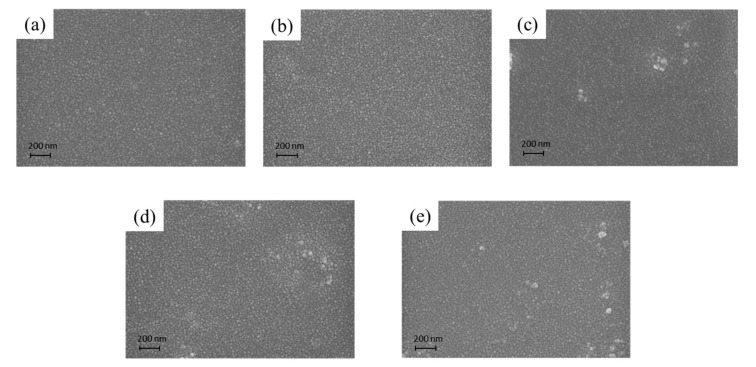
The scanning electron microscopy (SEM) analysis of the as-prepared nanohybrid silica polyacrylate film. (**a**) Without adding DFMA, (**b**) weight ratio of DFMA/MMA at 1:17, (**c**) weight ratio of DFMA/MMA at 1:8, (**d**) weight ratio of DFMA/MMA at 1:5, and (**e**) weight ratio of DFMA/MMA at 2:7.

**Figure 6:**
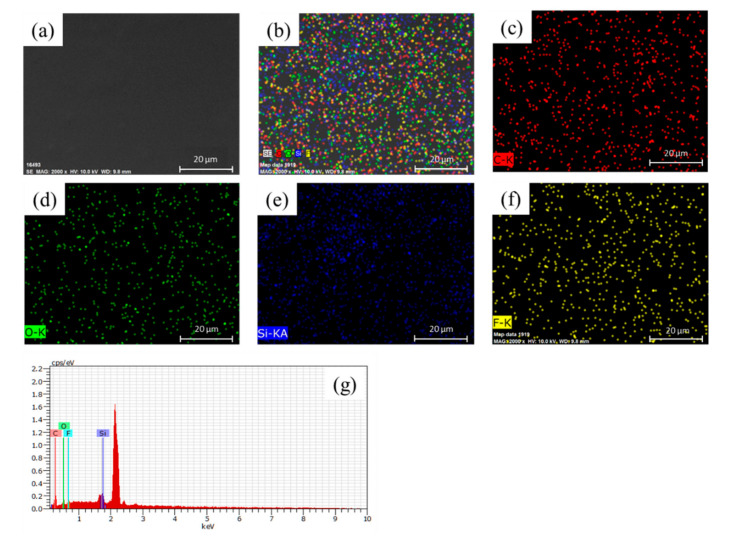
The mapping and EDS analysis nanohybrid silica polyacrylate film prepared by weight ratio of DFMA/MMA at 1:5. (**a**) SEM image of the as-prepared film, (**b**) mapping of C, O, Si, amd F elements on (**a**), (**c**) mapping of C element on (**a**), (**d**) mapping of O element on (**a**), (**e**) mapping of Si element on (**a**), (**f**) mapping of F element on (**a**), and (**g**) EDS analysis of the as-prepared film.

Figure 5 shows the SEM analysis of the as-prepared nanohybrid silica polyacrylate film. As seen from Figure 5a, when the polyacrylate material was prepared without the addition of DFMA monomer, the surface of the coating film only presented flat round particles and the nanoparticle arrangement was very neat. As the weight ratio of DFMA/MMA increases, a few micro-protruding particles can be examined on the surface, as shown in Figure 5b–e. This may be owing to the strong hydrophobicity of the fluorine segment contained in the DFMA monomer, resulting in the SiO_2_ particles agglomerating and becoming larger during the formation of fluorine-containing polyacrylate-SiO_2_ hybrid material [55]. Figure 6 shows the mapping and EDS analysis of the nanohybrid silica polyacrylate film prepared by a weight ratio of DFMA/MMA at 1:5. It can be seen that Si and F elements are also uniformly dispersed in the coating film.

**Figure 7:**
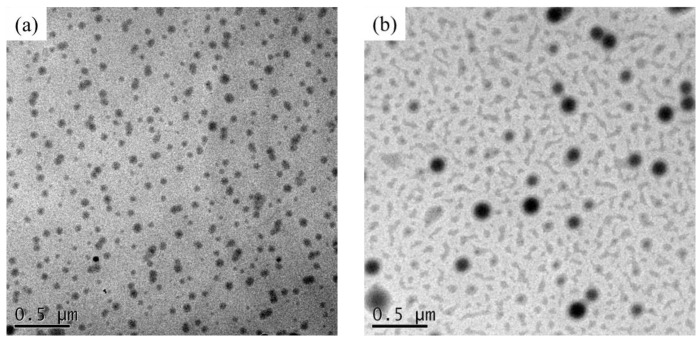
Transmission electronic microscopy (TEM) analysis of the as-prepared nanohybrid silica polyacrylate films. (**a**) Without adding DFMA and (**b**) DFMA/MMA weight ratio of 1:5.

Figure 7 shows the TEM analysis of the as-prepared nanohybrid silica polyacrylate films. It can be seen from the figure that the black spherical nanoparticle is SiO_2_, prepared by grafting the Si-coupling agent with TEOS through the sol–gel route in the polyacrylate-SiO_2_ hybrid material. The TEM image of the film was prepared without adding DFMA (Figure 7a) or a DFMA/MMA weight ratio of 1:5 (Figure 7b), respectively. It is clearly observed that, for the nanohybrid film obtained when the weight ratio of DFMA/MMA is 1:5, the particle size of the SiO_2_ in the hybrid material becomes larger, which is consistent with the results of Zhou et al. [57]. This may be because the greater hydrophilicity of Si sols renders them more open to becoming agglomerated during the sol–gel process, resulting in larger SiO_2_ particles, because the fluorine-containing segments in DFMA possesses strong hydrophobic properties [55]. This is in agreement with the aforementioned SEM analysis results.

Particularly, it is obvious from Figure 7b that the self-assembled black SiO_2_ nanoparticle (core) is coated with a gray area on the periphery, which contains the grafting of KH-570 to SiO_2_ (shell) in the fluorine-containing nanohybrid silica polyacrylate material [56]. In addition, a large irregular gray area outside the core–shell structural sphere may be a fluorine-rich copolymer separated from the non-fluorine copolymer, caused by the poor compatibility of fluorine-containing and non-fluorine copolymers [52]. The TEM analysis indicates that the polyacrylate–SiO_2_ coating film has a self-assembled structure regardless of the presence of DFMA. It may be that the Si-OH produced during the hydrolysis reaction of TEOS is a hydrophilic group, which is liable to self-assemble, with similar hydrophilic properties to the silicon coupling agent (KH-570), to form a core–shell structure in the nanohybrid silica polyacrylate material.

**Figure 8:**
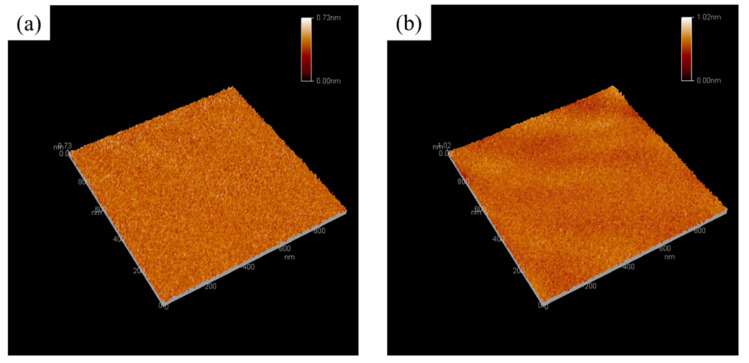
The atomic force microscopy (AFM) image of the as-prepared nanohybrid silica polyacrylate coating films. (**a**) Without adding DFMA and (**b**) DFMA/MMA weight ratio of 1:5.

Figure 8 shows the AFM image of the as-prepared nanohybrid silica polyacrylate films. The AFM image was taken in the scanning range of 1 µm × 1 µm. As the coating film was prepared without adding DFMA monomer, the root-mean-square (RMS) roughness of the coating film is 0.0566 nm (Figure 8a). Figure 8b shows the as-obtained film prepared with a DFMA/MMA weight ratio of 1:5; the RMS roughness of the surface of the nanohybrid silica polyacrylate film was 0.0638 nm, which was 12% more than the film obtained without the addition of the DFMA monomer. It can be speculated that the fluorine-containing polyacrylate material migrates to the interface of the film and air. Because of the addition of the DFMA-containing fluorine segment group in the acrylate polymer, the micro-phase separation occurs when the film is cured, thus increasing the surface roughness of the coating film [58,59].

### 3.4. Hydrophobicity and Transparent Properties of Nanohybrid Silica Polyacrylate Film

**Figure 9:**
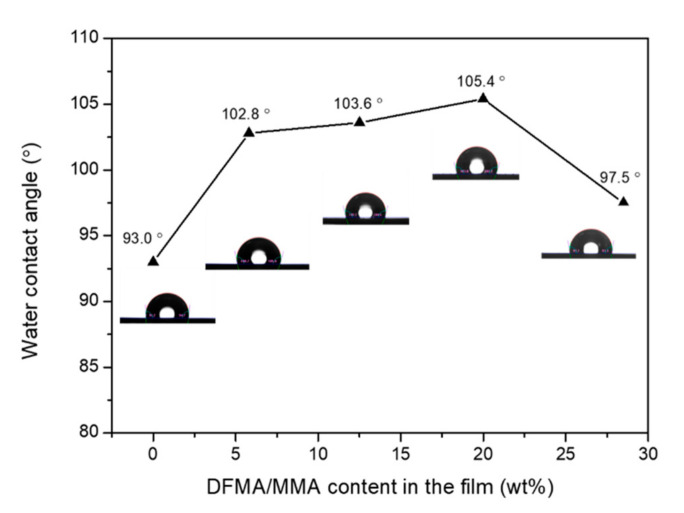
The water contact angle analysis of the as-prepared nanohybrid silica polyacrylate films prepared at different weight ratios of DFMA/MMA.

Figure 9 shows the water contact angle analysis of the as-prepared nanohybrid silica polyacrylate films prepared at different weight ratios of DFMA/MMA. Generally, when the water contact angle (θ) > 90°, the surface is hydrophobic; when the water contact angle (θ) < 90°, the surface is hydrophilic [2]. The figure shows that, when the weight ratio of DFMA/MMA changes from 0:9 to 1:5, the WCA of the as-prepared film increases from 93.0° to 105.4°. This is because the fluorine-containing segment group has low surface energy [55], leading to an increase in surface roughness and an increase in hydrophobicity. This is consistent with the results of the AFM analysis. However, when the weight ratio of DFMA/MMA increases to 2:7 in the raw materials of nanohybrid silica polyacrylate, the water contact angle decreases to 97.5° instead. As per the study of Zhou et al. [60], it has been proposed that, during the curing process of the higher fraction of fluorine-containing acrylate, the excessive fluorine atoms increase the steric effect in the structure of the copolymer and hinder the migration of fluorine atoms to the surface of the coating film. In addition, fluorinated polyacrylate containing large mole fractions of DMFA is excluded as a result of coagulum formation during polymerization [60]. Zhou et al. [60] also reported a similar variation tendency in the water contact angle as a function of the dosage of fluorine-containing acrylate monomer, hexafluorobutyl acrylate (HFBA), used to prepare polyacrylate resin.

**Figure 10:**
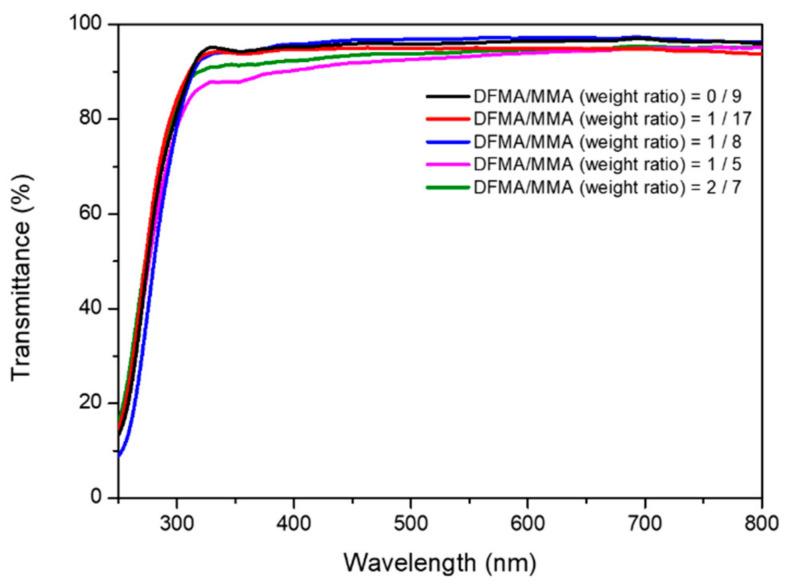
The UV–vis analysis of the polyacrylate coating films prepared from different weight ratios of DFMA/MMA.

The transparency of the as-prepared nanohybrid silica polyacrylate film can be characterized by ultraviolet–visible (UV–vis) spectroscopy. Figure 10 shows the UV–vis analysis of the polyacrylate coating films prepared from different weight ratios of DFMA/MMA. At various weight ratios of DFMA/MMA, the transparency of the as-prepared acrylate film decreased slightly. This could be explained by the fact that, when the ratios of DFMA/MMA increase, the roughness will increase and the transparency will decrease [1]. However, the transmittance is maintained at 89–97% for all of the as-prepared coating films in the visible light region (with a wavelength of 380 nm–760 nm). This result has a high transparency, with competitive capacity regarding the other hydrophobic coatings reported in the literature [61,62,63].

Combining the thermal stability, hydrophobic properties, and transparency of the as-prepared fluorine-containing nanohybrid silica polyacrylate material, the hybrid material possesses superb performances. This indicates that the coating material could be a very attractive practical solution in many areas, such as in applications for the coatings of 3C electronic products, vehicles, and the construction industry, especially in outdoor applications.

## 4. Conclusions

In this study, a fluorine-containing nanohybrid silica polyacrylate coating material was synthesized successfully by free radical polymerization and grafting with an organic–silicon compound. The influences of the DFMA/MMA weight ratios on the hydrophobic and transparent properties of the as-prepared coating film were discussed. The results indicated that the fluorine-containing group from DFMA improved the hydrophobic performance of the as-prepared polyacrylate-SiO_2_ nanohybrid film. The surface morphology of the as-prepared coating film exhibited spherical shape silica nanoparticle hybridizing with polyacrylic-based copolymer by grafting the coupling agent. The greater SiO_2_ particles exhibit an increase in surface roughness and enhance the hydrophobic properties of the as-obtained coating film, because the fluorine-containing segments in DFMA possess strong hydrophobic properties. Furthermore, the particle size of SiO_2_ in the material is larger when the weight ratio of DFMA/MMA is higher, because of the strong hydrophobic properties. The hydrophobic properties of the as-prepared coating film increase with the increase in the weight ratio of DFMA/MMA and reach their maximum values at 1:5. Furthermore, the increase in the weight ratio of DFMA/MMA lowers the hydrophobic performance. In addition, the thermal stability of the as-prepared nanohybrid silica polyacrylate material can be enhanced by the content of DFMA monomer that is added. Moreover, the surface morphology of the silicon coupling agent used to graft SiO_2_ with acrylate is spherical in shape and exhibits a core–shell structure, which increases the surface roughness and improves the hydrophobicity of the nanohybrid film. The as-prepared fluorine-containing nanohybrid silica polyacrylate film demonstrates a high transmittance of 89–97% in the visible light region.

## Figures and Tables

**Table 1:** The recipes of starting materials for the preparation of the nanohybrid silica polyacrylate coating materials.

First Stage (Free Radical Polymerization)								Second Stage (Sol–Gel Route)		
DFMA (g)	MMA (g)	DFMA/MMA (Weight Ratio)	DFMA/MMA (Molar Ratio)	2-EHA (g)	KH-570 (g)	THF (g)	AIBN (g)	TEOS (g)	HCl_(aq)_ (g)	THF (g)
0	9.0	0/9	-	1.0	0.05	40	0.02	1.0	1.5	5.0
0.5	8.5	1/17	1/68	1.0	0.05	40	0.02	1.0	1.5	5.0
1.0	8.0	1/8	1/32	1.0	0.05	40	0.02	1.0	1.5	5.0
1.5	7.5	1/5	1/20	1.0	0.05	40	0.02	1.0	1.5	5.0
2.0	7.0	2/7	1/14	1.0	0.05	40	0.02	1.0	1.5	5.0

## Data Availability

Data sharing is not applicable for this article.
